# A comparison between pulsed electric field and moderate electric field for their effectiveness in improving the freezing tolerance of rocket leaves

**DOI:** 10.1016/j.bbrep.2023.101515

**Published:** 2023-07-15

**Authors:** Imelda Christiana Nyoto, Federico Gómez Galindo

**Affiliations:** Department of Food Technology, Engineering and Nutrition, Lund University, PO Box 124, SE-22100, Lund, Sweden

**Keywords:** Electroporation, Stress, Cross-tolerance, Freeze-thaw survival

## Abstract

Two electrotechnologies: pulsed electric fields (PEF) and moderate electric field (MEF) in combination with vacuum impregnation of glycerol as cryoprotectant were used to increase the freezing tolerance of rocket leaves. Rocket leaves were treated with PEF using a nominal field strength of 1200 V/cm or MEF at different combinations of voltage and frequency. Leaves were then immersed in a glycerol solution at 32, 36 and 40% (w/v) under vacuum for 26 min. After this treatment, the leaves were allowed to rest for 3 days before they were frozen and thawed. Leaf survival was assessed at different time points after thawing with microscopic observations and wilting tests. When the viability of the leaves was assessed 5 min after thawing, 60–68% of the leaves in the batch survived. There was no difference in the levels of surviving leaves when PEF and the lowest-tested voltage used in MEF were tested. However, from the leaves surviving 5 min after thawing, approximately half of them die over a 24 h period after thawing.

## Highlights

• PEF, MEF and impregnated glycerol improve the freezing tolerance of rocket leaves.
• More than 60% of frozen leaves survive thawing but a fraction die during storage.
• From 25 to 37% of frozen leaves survive 24 h after thawing.

## 1 Introduction

Freezing and thawing techniques used for vegetables are successful for products with relatively strong cell walls such as green peas and carrots. Products with weak cell walls such as leafy vegetables become soggy after freezing and thawing, exuding liquid and losing much of their consumer appeal [1]. Efforts have been made to develop pre-treatment methods prior freezing to improve the quality of products that presently cannot be frozen without a considerable loss of quality and fresh-like characteristics [[2], [3], [4], [5]]. This paper will focus on the use of electrotechnologies as pre-treatment prior to freezing of rocket leaves.

Pulsed electric field (PEF) delivered to spinach leaves in such a way that they survived the consequent permeabilization of their cell membranes (reversible permeabilization) has been combined with vacuum impregnation (VI), a technology that used vacuum to impregnate a cryoprotectant in the intercellular spaces of the leaves. With the combination of these two treatments, cell viability was preserved after a freezing-thawing cycle, allowing cells to keep their turgor and the leaves to keep their fresh-like characteristics [6]. However, only 50% of the spinach leaves treated with this method survived the freezing and thawing cycle, making the method unsuitable for industrial practice [1].

A survivability of 89% was obtained by Demir et al. [1] when the authors exposed spinach plants to cold stress before harvesting. Changing the cultivation temperature from 20 to 5 °C during growth improved the survival of the harvested leaves after freezing and thawing when VI with trehalose and PEF were applied. Survival mechanisms triggered by this treatment have, to the best of our knowledge, not been described. The accumulation of T6P after VI with trehalose [7] in addition to stress responses to PEF might have influenced the capacity of the leaves to acquiree freezing tolerance.

In this study, an alternative to the use of PEF was tested on rocket leaves. Cell membrane permeabilization was achieved using moderate electric field (MEF). MEF involves a simpler, more direct application of electrical current (i.e. no capacitors, pulse forming networks, etc.) as an AC current (vs. DC in PEF) at considerably lower field strengths than PEF. The electroporation provoked by MEF can be either reversible or irreversible, depending on the strength of the electric field [8], which can be modified by changing the field intensity, frequency or treatment time. The lower filed strengths, longer treatment times of MEF may have a different metabolic effect in the leaves than those provoked by PEF, influencing their response to the freezing stress.

This paper aims at comparing the effect of the application of MEF and PEF in combination with VI with glycerol for the improvement of freezing tolerance of rocket leaves. Survival upon a freezing–thawing cycle was evaluated immediately and 24 h after thawing.

## 2 Materials and methods

### 2.1 Raw material cultivation

Rocket seeds (*Diplotaxis tenufoila* sp) were purchased from Enza Zaden, Copenhagen, Denmark under the market name PRUDENZIA F1. The seeds were planted in a greenhouse using a growing medium that consisted of plant soil (“Krukväxtjord med Lera & Kisel”, Weibulls Horto AB, Sweden) mixed with clay. The medium was mixed with perlite in a proportion medium:perlite 90:10. The seeds were planted in 56 × 25 × 6 cm (L x W x H) trays with perforations for drainage. The trays contained 2.5 kg mixed growing medium, with 7 × 3 planting holes per tray, 4–8 seeds per hole. During the growth of the plants, the position of the trays in the greenhouse was rotated every second day to ensure equal exposure of light of all trays.

The plants were watered daily. A fertilizer (Kristalon, Yara Vlaardingen B.V., Netherlands) with the composition N:P:K: 14:9:25 with MgO and traces of other minerals was used. The greenhouse was maintained at 16–18 °C and 45–55% RH with no additional lighting. The leaves were harvested after 51–58 days with the leaf blade size of 6.95 ± 0.73 cm of length and 2.74 ± 0.39 cm of width. The harvested leaves were kept in a humid box and used for experiments the same day of harvest. The leaves were rinsed with tap water before used in the experiments.

### 2.2 Treatments

#### 2.2.1 Electrical treatments

**Fig. 1:**
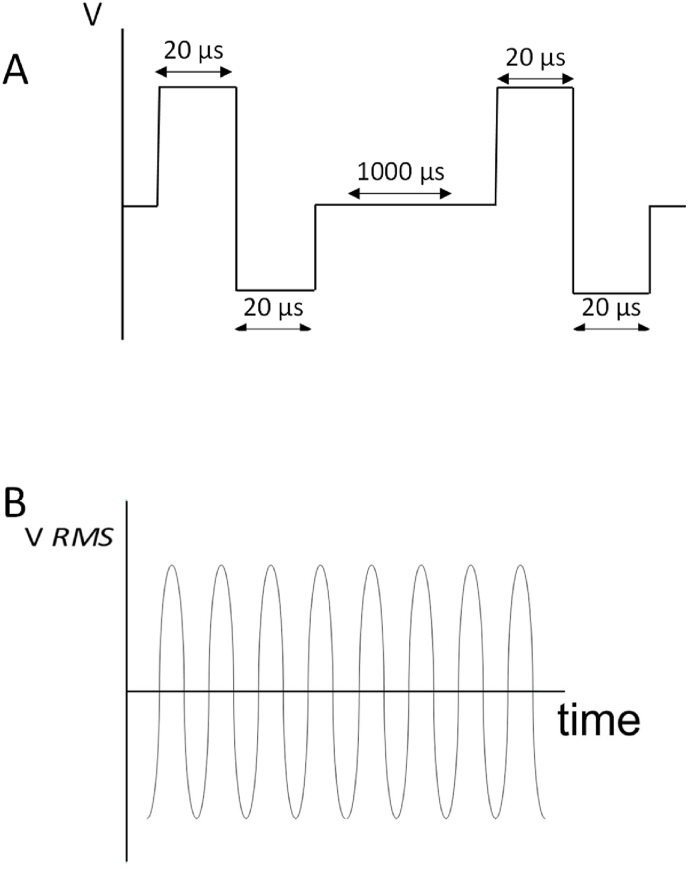
Schematic representation of the applied electrotechnologies, showing squared pulses for the application of bipolar PEF (A) and sinusoidal, electric field wave form for MEF (B).

Leaves were placed in an electroporation chamber with 5 mm gap between two parallel stainless-steel electrodes (50 mm wide and 100 mm long). The gap between the electrodes was filled with a diluted NaCl solution with a conductivity of 250 μS. The chamber was then connected to a pulse generator (ADITUS AB, Lund, Sweden) for PEF treatments or to an AC Power source (3000 VA, BK Precision, CA, USA) for MEF treatments. The temperature increment during PEF and MEF treatments were less than 2 °C. The electric pulses were monitored using a handheld oscilloscope (Fluke 1238 industrial scope meter, Fluke, USA). Electroporation protocols were delivered by combining the parameters in the range shown in Table 1. A schematic showing the difference between PEF and MEF treatments is shown in Fig. 1. The parameters providing reversible electroporation to leaves were determined in preliminary experiments with fluorescence microscopy, using propidium iodide as an electroporation indicator and fluorescein diacetate as a cell viability indicator, as described by Ref. [9]. At least three batches of 25 leaves each were used for each of the treatments.

**Table 1:** Range of parameters used for the studied electrical treatments.

Protocol	Voltage (V/cm)	Pulse width (μs)	Pulse space (μs)	Number of pulses	Frequency (Hz)	Treatment time (ms)	Polarity
PEF	1200	20	1000	500	–	–	Bipolar^a^
MEF A	28^c^	–	–	–	50	2000	Bipolar^b^
MEF B	156^c^	–	–	–	50	600	Bipolar^b^
MEF C	156^c^	–	–	–	600	600	Bipolar^b^

a The total length of the pulse was divided into half positive and half negative.

b Bipolar sinusoidal.

c Value calculated from the Vrms provided by the equipment.

#### 2.2.2 Vacuum impregnation

After the PEF treatment, the leaves were immediately immersed in the glycerol cryoprotectant solution and placed into a chamber for VI. Three hypertonic glycerol solutions were used: 32% w/v (G1), 36% w/v (G2) and 40% w/v (G3). The VI treatment was carried out at 20 °C in a chamber connected to the automatic vacuum-controlled system (AVSC. S.I.A., Bologna, Italy) described by Ref. [10]. A stepwise protocol with a minimum absolute pressure of 90 mbar was chosen for 26 min. This duration comprised a gradual increase of the vacuum for 10 min, a holding time of 1 min, and a gradual release of the vacuum for 15 min.

#### 2.2.3 Combination of treatments

**Fig. 2:**
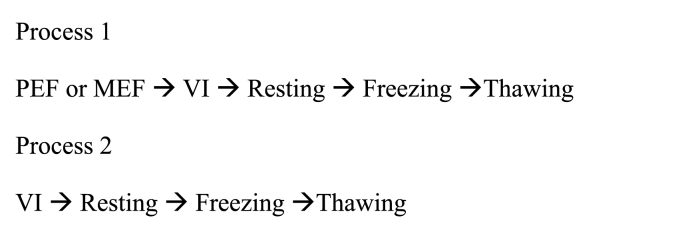
A combination of treatments for improving the freezing tolerance of rocket leaves. Each unit operation was performed as described in the Materials and methods section.

Rocket leaves were treated with a combination of PEF and VI prior to freezing and thawing. The procedures and their corresponding processing steps are shown in Fig. 2, where leaves were treated with two different processes.

#### 2.2.4 Resting

After VI in processes 1 and 2 (Fig. 1), the leaves were stored in a plastic container (24 cm × 17 cm x 5.5 cm) for resting. Wet tissue paper was placed at the bottom of the plastic container. A plastic net was placed on the wet tissue to avoid direct contact between the leaves and the wet paper. The plastic container was then stored in the darkness for 3 days at 3.2 °C–5.6 °C and 94–99.9% RH (as monitored. with a USB standard ST-171 temperature and humidity data logger, Clas Ohlson, Sweden). The resting period was stablished in preliminary experiments where rocket survival after 1, 2 and 3 days were tested with a fix concentration of the cryoprotectant and the described PEF treatment. A 3-days resting period was chosen as no survival was detected upon a freeze-thaw cycle when the leaves were rested for 1 and 2 days.

#### 2.2.5 Freezing and thawing

After resting, the leaves were gently blotted with a soft tissue to remove excess of liquid. The leaves were then frozen in a chest freezer (Electrolux, Italy) at −22 °C and kept in the same freezer for 24 h. The frozen leaves were thawed on a tissue paper at room temperature for 5 min. The thawed leaves were then stored in an humid container in the darkness under refrigeration for further analysis.

### 2.3 Analysis

#### 2.3.1 Microscopic observation of leaf surface electroporation and viability of the tissue

To investigate the effect of the different protocols on the electroporation of the cells on the leaf surface, propidium iodide (PI) was used as an electroporation indicator, as described by Ref. [9]. PI (Sigma-Aldrich, USA, λex = 535 nm, λem = 617 nm) was used to stain the nucleus of the cells upon permeabilization. Three rocket leaves were placed in the electroporation chamber filled with 250 μM PI solution in 10 μM PBS solution with a conductivity of 250 μS/cm before being subjected to electrical treatment. Treated leaves were rinsed with running tap water and gently patted dry with absorbent paper prior to microscopic examinations. The observation was conducted using a fluorescence microscope (Elipse TieU, Nikon, Japan) at 10X magnification. The images of the samples were taken with a digital camera (digital sight DS-Qi1Mc, Nikon Co., Japan).

Survival of the samples was investigated using fluorescein diacetate (FDA; Sigma-Aldrich, USA, λex = 492 nm, λem = 517) as described by Ref. [9], which was used to stain viable cells. FDA stock solution (12 μM) in acetone was prepared and stored in the dark at 4 °C. On the day of the experiment, 16 h after PEF or MEF treatment, the stock solution was diluted with deionized water to the final concentration of 12 × 10−4 μM. Viable cells were easily identified under the microscope by bright fluorescence [9].

#### 2.3.2 Electrical resistance

An uncut rectangular portion of a whole rocket leaf was placed in between two flat, parallel stainless-steel electrodes (9 × 5 mm) with wet (10 μl of a NaCl solution with 250 μS) filter paper (MN 713, Macherey Nagel, Germany) between the sample and the electrodes. The thickness of the filter paper was 0.28 mm. The electrodes were slightly squeezed with a plastic clamp to ensure that the entire area of the leaf sample was attached to the electrodes. The sandwich structure of electrodes and sample was placed in a Styrofoam box with wet paper at the bottom and connected with cables to the impedance analyser (4192A LF impedance analyser, Hewlett Packard, USA).

The resistance measurement was taken and recorded straight after the sample was attached to the analyser at the frequency of 50 kHz and repeated 1 min and 1 h after the application of PEF or MEF. Untreated leaves were used as control to monitor possible leaf weight changes during the storage of the sandwich.

#### 2.3.3 Viability analysis

**Fig. 3:**
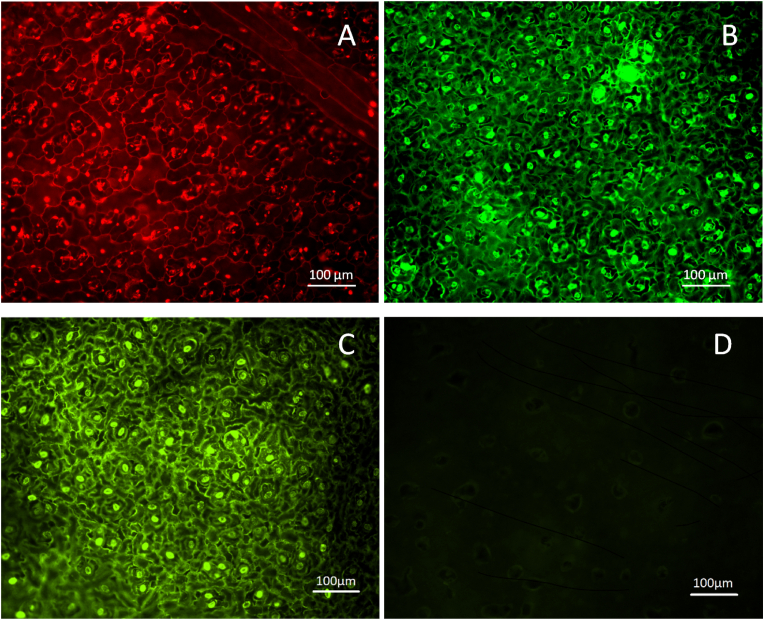
Representative micrographs from microscopic observations of rocket leaf surface. A: Homogeneous surface permeabilization detected by PI, B: Viable cells seen after PEF or MEF protocols, as shown by FDA fluorescence, C: Viable cells seen in a fresh, untreated rocket leaf, D: Dead cells do not show FDA fluorescence.

After thawing, the turgidity of the treated rocket samples was evaluated as described by Ref. [1]. The centre of the leaf was placed on a small bamboo rod (3 mm diameter) and observed whether the leaf would bend or not. The leaves that did not completely wilt after thawing were counted as surviving leaves. The surviving leaves were placed in a plastic box with wet paper at the bottom and stored under refrigeration at 4 °C (±2 °C). The percentage of surviving leaves from a total of 25 leaves on selected treatments was evaluated at different time points after thawing: 5 min, 30 min, 1 h, 2 h, 5 h and 24 h. Randomly selected surviving leaves were additionally treated with FDA as described above to verify the viability of the cells in the leaves. Each leaf was sampled in two places for observation of viability under the microscope, at the centre and at the top (Fig. 3D and E).

### 2.4 Statistical analysis

Statistical significance (p < 0.05) of the treatments was tested by means of one-way-ANOVA using MINITAB 17 software (Minitab Inc., State College, PA, USA). The Tukey–Kramer multiple comparison test was used to evaluate differences between treatments.

## 3 Results

### 3.1 Evaluation of reversible electroporation

The electroporation of the tissue by the applied reversible PEF and MEF conditions is demonstrated by the penetration of propidium iodide in the cells, staining their nucleus (Fig. 3A). The long-term survival after the application of the PEF or MEF treatments is shown by the fluorescence of the cells stained with FDA (Fig. 3B). FDA vital staining of a fresh leaf is shown in Fig. 3C (positive control) and FDA staining of a fresh leaf that was frozen and thawed is shown in Fig. 3D (negative control). Since dead cells cannot hold the FDA molecules, they do not show fluorescence, while the living cells appeared as bright green.

### 3.2 Recovery of electrical resistance after electroporation

**Fig. 4:**
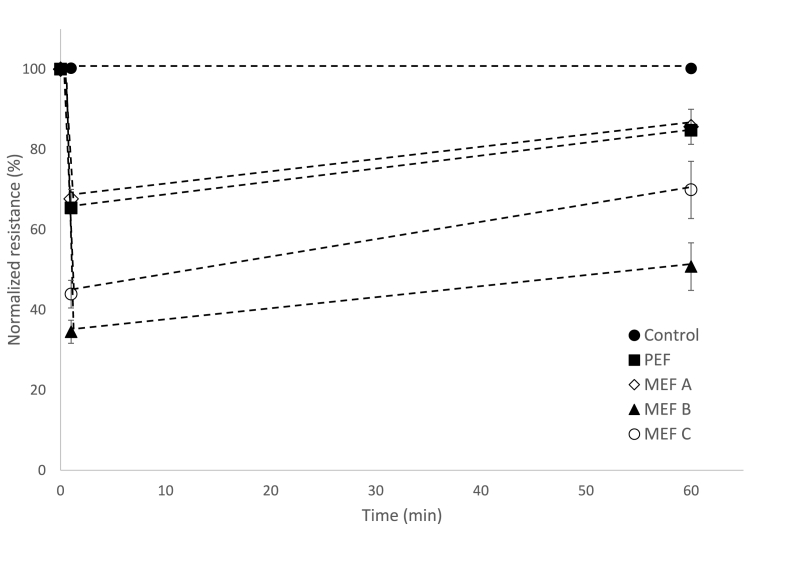
The change on tissue resistance as a function of the time after the application of different electrical treatments. Reported are average and standard deviation of at least 12 measurements. Values have been normalized so that the pre-pulsation resistance equals to 100.

The resistance of the leaves, measured 1 min after the MEF or PEF treatments, showed a sharp decrease (Fig. 4), where MEF treatments with the highest applied voltage provoked the highest decrease of resistance and, therefore, provoked the strongest effect on cell membrane permeability.

The measurement of resistance 1 h after the pulse, showed more than 80% recovery of the initial resistance value of leaves treated with PEF and with the lowest-voltage MEF treatment.

### 3.3 Survival of leaves after freezing and thawing

**Fig. 5:**
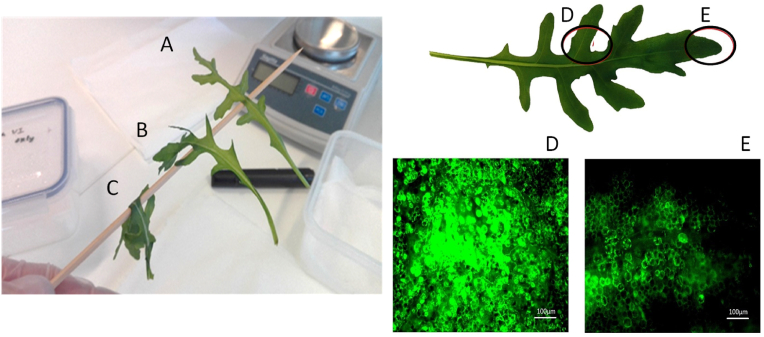
Survival of rocket leaves upon a freezing and thawing cycle. Left panel: Representative results from the wilting test. The leaves were treated, frozen and thawed as described in the Materials and methods section. After thawing, the leaves were suspended on a bamboo rod A: fresh, untreated leaf, B: surviving leaf after freezing and thawing, C: non surviving leaf after freezing and thawing. Right panel: Sampling of a surviving rocket leaf after a freezing and thawing cycle. D: Representative micrograph showing viable cells after staining with FDA. This sample corresponds to a tissue sampled from the middle of the leaf, E: Representative micrograph showing FDA staining of viable cells on tissue sampled from the top of the leaf. Bright areas represent viable cells, dark areas represent dead cells.

Fig. 5B show a typical result obtained from the wilting test after the leaves were frozen and thawed. It can be seen that the leaf is typically not fully turgid, as the fresh leaf (Fig. 5A) or totally wilted as the negative control (Fig. 5C). A further investigation with FDA on treated, surviving leaves, reveals that the central parts of the leaf contain more viable cells (Fig. 5D), which contribute to the turgidity of the tissue, while the top of the leaf has a mix of viable and non-viable cells (Fig. 5E), which would explain the bending of that part of the tissue on the wilting test (Fig. 5B).

**Fig. 6:**
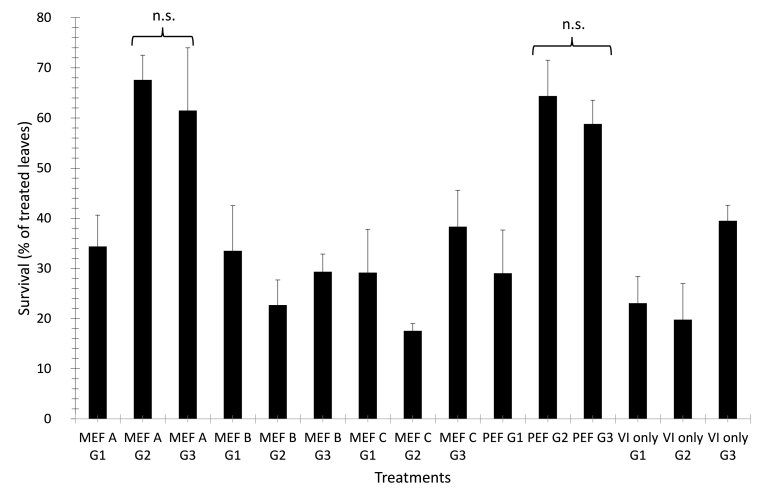
Percentage of surviving rocket leaves after a freezing and thawing cycle. The evaluation of survival was done 5 min after thawing was completed. The leaves were treated as described in “process 1” and “process 2” (Fig. 2), using glycerol at different concentrations (G1, G2 and G3). Reported are average and standard deviations of at least 75 leaves. n.s: not significant differences.

The percentage of surviving leaves, 5 min after thawing, for the different treatments is reported in Fig. 6. The MEF treatment using the lowest voltage and the two highest concentrations of glycerol as well as the PEF treatment using the same concentrations of the cryoprotectant show the best survival results with more than 60% of the leaves surviving the treatment. No significant differences were obtained between the two highest glycerol concentrations.

**Fig. 7:**
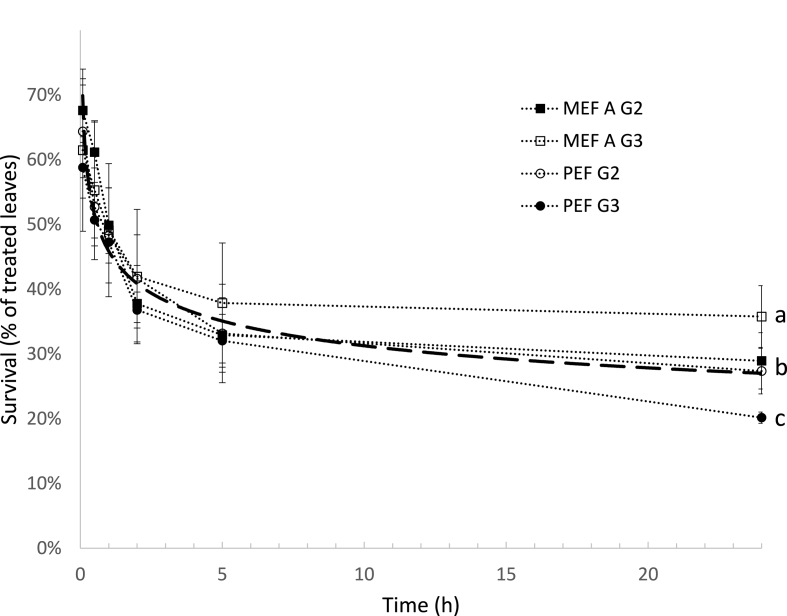
Post-thawing survival of rocket leaves pre-treated with PEF and the MEF protocol using the lowest voltage (Table 1) and subjected to VI with two different concentrations of glycerol (G2 and G3) prior to freezing and thawing (dotted lines). The dashed line represents the fitting of the data to a power law model (y = 0.4588x^−0.167^; R^2^ = 0.8859). Different letters next to the error bars indicate significant differences.

The treatments with the highest survival results were chosen for a further evaluation of the post-thawing life of the leaves. Fig. 7 shows that the percentage of surviving leaves decreases over time with a drastic decrease (>40%) 24 h after thawing. However, most of the decrease of survival occurred 5h after thawing with only slight changes in the next 19 h, except for the PEF G3 treatment, where a further decrease of survival was detected. The data for all treatments were fitted into a power law model and the result is reported in Fig. 7 (dashed line).

## 4 Discussion

Increased cryoprotection of spinach leaves by the application of the combination of pulsed electric field and the vacuum impregnation of trehalose as a cryoprotectant has been thoroughly documented [1,6]. In the case of the present study, despite a slight change of taste, the method proved to increase the freezing tolerance of rocket leaves, using glycerol as cryoprotectant. This result was also achieved using MEF to provoke reversible electroporation (Fig. 5). Interestingly, in the case of MEF, the rocket tissue could be electroporated with rather low applied voltage (Table 1) achieving comparable cryoprotection results with those obtained with PEF.

The disruption of the natural balance of the cells when pores are opened *per se* provoke stress responses that may be responsible for the increased tolerance. The results presented here suggest that the treatment time and the frequency are also important factors influencing survival to freezing and thawing. These factors may influence the recovery of the tissue form the elecropermeabilization of the cell membranes (Fig. 3) and, therefore, the capacity to tolerate a second stress (freezing) [1]. The time between the pulses may or may not be enough for the cell membrane damage caused by the treatment to be repaired and the response of the cells would depend on whether they “feel” the pulses as separate events or not [11].

After the initial sharp decrease, cell recovery after PEF shows a progressive increase of the tissue resistance (Fig. 3). This increase may be caused by the taking up of leaked ions, a process that is ATP-dependent [12]. VI is then performed on a tissue that is recovering, which may facilitate the absorption of the cryoprotectant inside the cells, contributing to the detected increase of freezing tolerance. However, independently on what could be the mechanism behind this cryoprotection, a fraction of all treated leaves dies within minutes after thawing (Fig. 6).

Despite uncertainties on the effect of electropermeabilization parameters on cells, there is overwhelming evidence in the literature showing that, upon permeabilization, reactive oxygen species (ROS) are produced [13,14]. Depending on the pulsing conditions, the production of ROS has been reported to increase sharply on intact maize cells for 5–7 min after the application of the pulse until reaching a plateau [14]. In maize cells, the generation of ROS could be detected even 1 h after pulsing [14]. It is, however, expected that the long resting time between the application of PEF or MEF and the freezing of the leaves should be accompanied by a decay on ROS production.

Cells surviving electropermeabilization will be subjected to a second event leading to oxidative stress: the freezing-thawing cycle. Oxidative stress caused by uncontrollable production of harmful ROS would partially contribute to cause injury upon a freezing-thawing cycle [15]. Excessive ROS production may induce oxidative modification of cellular macromolecules leading to cell death [16]. After thawing and, independently on the electroporation method or pulse parameters used as pre-treatment, there are three fractions of leaves (i) leaves that permanently survive the freezing-thawing cycle 24 h after thawing (“survivors”), (ii) leaves that die within the first 2 h after thawing (“fast dying”) and (iii) leaves that take longer time to die (“slow dying”) (Fig. 6). Survivors may have gained resistance to the damage thanks to the cross-tolerance provoked by the previous PEF or MEF application. Protection against freezing stress is the result of cross talk between stress signalling pathways [1]. Fast and slow dying leaves, pretreated with PEF and low-voltage MEF, seemed to recover from the electric treatment (Fig. 3) but they cannot survive freezing-thawing. This unpredictable survival pattern by leaves from the same batch, treated in the same way has also been reported for spinach leaves [1].

As a result of the above-mentioned considerations, few questions are open for further investigation: What metabolic factors and/or stress responses would be the key to “decide” if the PEF or MEF pre-treated leaves would survive freezing-thawing or not? Can stress-induced cross tolerance be influenced by the choice of electroporation parameters? Is there any metabolic marker that can be used for the optimization of processing parameters that can enhance freezing survival? The answer to these questions would allow to acquire vital knowledge for finding the most appropriate processing conditions for a successful increase of the freezing tolerance in rocket leaves.

## 5 Conclusion

This study provides evidence that the freezing tolerance of rocket leaves improves when either PEF or MEF are applied in combination with the impregnation of the cryoprotectant glycerol as pretreatment prior to freezing. The effect on freezing tolerance was dependent on the chosen parameters for electroporation as well as the concentration of glycerol. More than 60% of the treated rocket leaves survived a freezing and thawing cycle under the most successful processing conditions. However, during short-term frozen storage, the survival of the leaves decreased drastically.

## Declaration of competing interest

The authors declare that they have no known competing financial interests or personal relationships that could have appeared to influence the work reported in this paper.

## Data Availability

No data was used for the research described in the article.
